# Temporomandibular joint synovial chondromatosis

**DOI:** 10.1016/S1808-8694(15)30591-7

**Published:** 2015-10-19

**Authors:** Bruno De Santi Bonatti, Lucas Gomes Patrocinio, Sérgio Antonio Araújo Costa, José Mariano Carvalho Costa, José Antonio Patrocinio

**Affiliations:** 1 MD. ENT Resident – University of Uberlândia Medical School.; 2 ENT. MD. University of Uberlândia Medical School.; 3 Dentistry Student.; 4 MSc. Maxillo-Facial Surgeon, Department of Otorhinolaryngology, University of Uberlândia Medical School.; 5 Full Professor. Head of the Otorhinolaryngology Department – University of Uberlândia Medical School. Otorhinolaryngology Department – Federal University of Uberlândia Medical School -Faculdade de Medicina da Universidade Federal de Uberlândia, Uberlândia, Minas Gerais, Brasil.

**Keywords:** synovial chondromatosis, dental occlusion, temporomandibular joint disorders

## INTRODUTION

Synovial chondromatosis (SC) is a rare, benign cartilaginous metaplasia that affects the joints, usually only one joint^1^, ^2^.

Chondromatosis is a hard to diagnose disease because of its somewhat vague symptoms, especially those in the temporo-mandibular joint (TMJ), which does not fit those from the most prevalent disease sites (knee, elbow and shoulder). There is only one case of TMJ chondromatosis in the Brazilian Literature^2^.

The goal of the present investigation is to report a case of TMJ chondromatosis, discussing its pathological aspects and treatment.

## CASE REPORT

**Figure 1:**
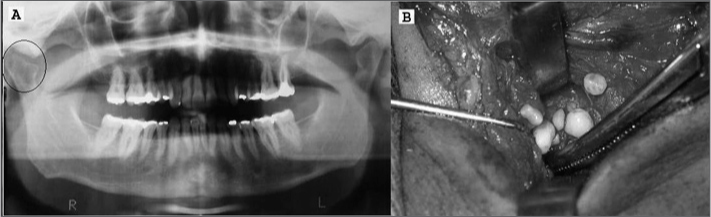
Panoramic mandible x-ray showing an alteration (Circle) in the right TMJ (A) and surgical removal of the synovial chondromatosis (B).

C.F.V., 40 years of age, male, presented pain in his left TMJ, mild facial asymmetry, crepitation, constrained mouth opening, without association with trauma or joint disease. A panoramic x-ray of his TMJ showed an alteration in his left TMJ region (Figure 1A). The CT scan showed an abnormal growth in the supero-posterior compartment of his left TMJ. An incisional biopsy and subsequent pathology exam suggested a diagnosis of chondroma.

Surgery was performed in order to remove the tumor through a pre-auricular incision. The tumor was located more medially to the joint capsule (spheno-maxillary space) and 108 nodules of varied sizes were removed (Figure 1B). The articular capsule was then sutured with absorbable wire and the skin was sutured with non-absorbable wire with simple stitches.

There were no intraoperative or postoperative complications. The pathology report described bony tissue with trabeculae and intra-trabecullar spaces without alterations, followed by cartilaginous tissue nodule formations, with calcifications, without cellular atypias, supported by connective tissue, matching signs of chondroma. The patient is under follow up for 4 years now and has had no recurrences.

## DISCUSSION

SC affects mainly the joints of long bones such as: knees, elbows, shoulders and, very rarely, the TMJ1, it is more common in the second and third decades of life. However, when it affects the TMJ, it is more common in women (1.5:1) during their fourth and fifth decades of life. It is rare in children^3^. It affects more often the right TMJ (4:1)^1^.

Both the primary and secondary forms of the disease have been described. In the primary, the etiology is still unknown; however, most researchers believe it to be associated with embryologic disorders, that is, a cartilaginous metaplasia of synovial tissue remains. The secondary form is associated with trauma, infection or articular disease, such as inflammatory and non-inflammatory osteochondritis and arthroplasty^1^.

Diagnosis is based on clinical manifestations and complementary tests. The main symptoms include: pain, joint swelling, stiffness, crepitation, and functional limitations, usually progressive and of long duration. Often times it can be erroneously treated as an internal TMJ disorder. A tumor in the parotid area can also be misdiagnosed as a parotid tumor^4^.

Histology is essential in these cases in order to differentiate an SC from a chondrosarcoma. Thus, no necrosis, myxoid cartilage, mitotic activity and spindle-like cells point toward a benign behavior^3^.

Treatment for SC is surgical and objective, removing all the loose bodies. Depending on disease progression, there can be the need to smoothen the joint surface, partial or total removal of the synovial membrane and carry out a discectomy^2^.

## CONCLUSION

Differential diagnosis of TMJ lesions is not always easy, and the definitive diagnosis is only reached after the pathology exam. SC has vague signs and symptoms, however must be always considered when we have a patient with otalgia and TMJ pain.
